# Poor Correlation Among Metal Hypersensitivity Testing Modalities and Inferior Patient-Reported Outcomes After Primary and Revision Total Knee Arthroplasties

**DOI:** 10.1016/j.artd.2022.09.016

**Published:** 2022-10-31

**Authors:** Daniel N. Bracey, Vishal Hegde, Roseann Johnson, Lindsay Kleeman-Forsthuber, Jason Jennings, Douglas Dennis

**Affiliations:** a Department of Orthopaedic Surgery, University of North Carolina, Chapel Hill, NC, USA; b Department of Orthopaedic Surgery, The Johns Hopkins University, Baltimore, MD, USA; c Colorado Joint Replacement, Denver, CO, USA; d Department of Mechanical and Materials Engineering, University of Denver, Denver, CO, USA; e Department of Orthopaedics, University of Colorado School of Medicine, Denver, CO, USA; f Department of Biomedical Engineering, University of Tennessee, Knoxville, TN, USA

**Keywords:** Metal hypersensitivity, Metal allergy, Total knee arthroplasty, Skin patch test, Lymphocyte transformation test

## Abstract

**Background:**

Metal allergy testing may influence clinical decision-making for patients undergoing a total knee arthroplasty (TKA). Limited data were found to examine the consistency of available testing modalities. This study compares different metal allergy test results and clinical outcomes after primary and revision TKAs in patients with and without metal hypersensitivity.

**Methods:**

Primary (n = 28) and revision (n = 20) TKA patients receiving hypoallergenic implants for metal allergies diagnosed by skin patch testing (SPT), lymphocyte proliferation testing (LPT), or lymphocyte transformation testing (LTT) were retrospectively reviewed. The agreement between tests was assessed by percentage and kappa statistic within patients who used multiple testing modalities. Postoperative clinical outcomes of these patients were compared to those of patients without metal hypersensitivity matched by age (±5 years), body mass index (±5), gender, and follow-up duration (±2 years).

**Results:**

SPT and LPT showed weak agreement for nickel and minimal agreement for cobalt. SPT and LTT showed minimal agreement for nickel; weak agreement for titanium, bone cement, vanadium, and zirconium; but strong agreement for chromium and cobalt. LPT and LTT agreement was weak. Compared to matched controls, metal hypersensitivity patients undergoing primary TKAs with hypoallergenic implants experienced less improvement in Knee Society Scores, Veterans RAND 12 physical component scores, and range of motion. Patients undergoing revision TKAs for multiple indications including metal hypersensitivity had worse clinical outcomes with significantly worse improvements in Knee Society functional scores compared to matched controls.

**Conclusions:**

Metal allergy tests produce conflicting results. Hypersensitivity patients may experience inferior clinical outcomes even with hypoallergenic implants. Clinician awareness may influence the choice of testing and improve preoperative counseling of this patient population.

## Introduction

Metal hypersensitivity complicates clinical decision-making in patients preparing to undergo a total knee arthroplasty (TKA). Metal hypersensitivity is relatively common in the general population, with a reported prevalence of 10%-15%, [[1], [2], [3]] but their relevance to clinical outcomes after a TKA is controversial in arthroplasty literature. Metal hypersensitivity syndromes remain a diagnosis of exclusion given the lack of objective diagnostic tools with insufficient sensitivity and validity [1,4]. While a variety of diagnostic tests exist, contact skin patch testing (SPT) and lymphocyte blood tests remain the most common testing modalities [3,[5], [6], [7], [8]]. In patients with self-reported metal hypersensitivities or positive contact allergy test results, the indications for the use of hypoallergenic metal implants are currently unclear with inconsistent clinical results [[9], [10], [11], [12], [13], [14]].

The immune response to arthroplasty components is a delayed type IV hypersensitivity cell-mediated response from activated lymphocytes and macrophages [1,2,5]. The validity of the SPT has been questioned because this test assesses the Langerhans cells' reaction to a topical metal allergen using a subjective grading scale. To accurately assess the potential metal hypersensitivity in a patient, it is likely that more than 1 testing modality is necessary, such as the triple assay technique originally described by Hallab et al. [15]. Lymphocyte transformation testing (LTT) is an in vitro, quantitative assessment of the patient’s lymphocyte stimulation by metal allergens in a solution [5]. This test is believed to have greater sensitivity that more closely simulates the body’s reaction to arthroplasty components which is mediated through the activation of T-lymphocytes [1,2,7,13]. Limited data have been presented on patients with results from multiple metal allergy testing modalities, [6,9] and to our knowledge, only 2 studies have reported a statistical analysis on the concordance between the different testing modalities on individual patients [7,16].

Studies reporting clinical outcomes after TKAs with hypoallergenic implants for confirmed or suspected metal hypersensitivities have produced conflicting data [5,[12], [13], [14],[17], [18], [19]]. Patients with hypersensitivity confirmed by contact SPT have been shown to have equivalent outcomes to patients with negative SPT receiving the same TKA implants [14]. Hypersensitive patients have also been shown to do worse after TKAs even if hypoallergenic implants were used [13,17].

The current study investigates the concordance of metal hypersensitivity testing modalities by comparison of different test results within individual patients. Clinical relevance of metal hypersensitivity testing is studied by comparison of clinical outcomes after primary and revision TKAs in patients with metal hypersensitivity receiving appropriate hypoallergenic components against outcomes in matched nonhypersensitive patients receiving standard components.

## Material and methods

With institutional review board approval, the medical records of all patients undergoing primary and revision TKAs using hypoallergenic components were retrieved from our institution’s prospective, longitudinally maintained total joint arthroplasty database in order to capture a large sample of patients expected to have metal hypersensitivity testing results. All retrieved records were reviewed to identify patients with more than 1 type of documented metal hypersensitivity test. Metal hypersensitivity tests included for study consisted of contact SPT, LTT, lymphocyte proliferation testing (LPT), and memory lymphocyte immunostimulation assay (MELISA). Tested metal allergens included nickel, cobalt, chromium, titanium, copper, vanadium, zirconium, aluminum, and molybdenum. Within each patient, the different test results for each metal were compared. The agreement between the different testing modalities was assessed with percentage agreement and kappa statistic.

Clinical outcomes in patients with metal hypersensitivities undergoing primary and revision TKAs by 4 fellowship-trained arthroplasty surgeons between 2011 and 2019 were retrospectively reviewed. Metal hypersensitivities were confirmed by metal allergy testing, and patients with self-reported metal hypersensitivities were excluded from the study if no documented test results were available. Outcomes were compared to a matched patient cohort without metal hypersensitivity undergoing primary and revision TKAs. Patients were matched by age (±5 years), gender, body mass index (±5), and postoperative follow-up duration (±2 years). Patients with metal hypersensitivities received appropriate hypoallergenic TKA implants while patients without metal hypersensitivities received standard nonhypoallergenic TKA implants. Metal hypersensitivity was the primary indication for revision TKAs in patients with confirmed metal hypersensitivities. The hypoallergenic implants used in our practice were at the discretion of the surgeon and included a mixture of titanium components, titanium-nitride-coated implants, all-polyethylene tibial components, and oxinium oxidized zirconium implants. Clinical outcomes assessed included preoperative and postoperative range of motion (ROM), Knee Society Scores (KSS), KSS function score, and Veterans RAND 12 mental and physical component scores.

The strength of the agreement between different metal hypersensitivity testing modalities was interpreted from the kappa statistic value [20]. Clinical outcomes data with continuous variables were compared between groups using student t-tests. Statistical significance was determined using α < 0.05.

## Results

Forty-eight patients (n = 28 primary TKA, n = 20 revision TKA) who had documented metal hypersensitivity testing and 35 patients who had multiple types of testing were identified (Table 1). Of these 48 patients, the most common testing modality used was contact SPT (n = 40), followed by LPT (n = 29), LTT (n = 15), and MELISA (n = 2). Twenty-nine patients had both SPT and LPT testing, 11 patients had both SPT and LTT testing, 6 patients had SPT, LTT, and LPT testing, and 2 patients had all 4 testing modalities. All SPT and LPT tests were performed at a single institution (National Jewish Health, Denver, CO), all LTT tests were performed at a single laboratory (Orthopedic Analysis, Chicago, IL), and the MELISA testing was performed at a single lab as well (Pharmasan Labs, Osceola, WI). SPT assessed the largest sample of different metals, most commonly nickel, cobalt, and chromium. LPT most commonly assessed nickel, followed by cobalt, and chromium was only assessed in 1 patient. LTT testing assessed the same 9 metals in all 15 cases. MELISA assessed the same 7 metals in 2 patients. The most frequent combination of testing observed for a correlation analysis was SPT and LPT. The agreement between SPT and LPT tests was weak for nickel in 28 patients (71% matching results, κ = 0.43) and for cobalt in 17 patients (58% matching results, k = 0.17, n = 17). There were 11 patients with both SPT and LTT tests for various metals. The agreement between SPT and LTT tests was minimal for nickel (54% matching results, k = 0.09, n = 11) but strong for cobalt and chromium (91% matching results, k = 0.8, n = 11). There were 6 patients with both LPT and LTT tests that had weak agreement (67% matching results, k = 0.5, n = 6). There were only 2 patients with MELISA test results in addition to another test which precluded the correlation analysis.

**Table 1:** Patient metal allergy testing.

ID	Patch testing										LPT			LTT									MELISA						
	Al	Cem	Co	Cr	Cu	Mo	Ni	Ti	V	Zr	Co	Cr	Ni	Al	Cem	Co	Cr	Mo	Ni	Ti	V	Zr	Al	Co	Cr	Mo	Ni	Ti	V
1	−	−	−	−	−	−	−	−	−	−	+		−																
2	−	−	+	−	+	−	+	−	−	−	−		+																
3	−	−	−	−	−	−	+	−	−	−			+																
4			+				+				+		+																
5	−	+	−	−	−	−	−	−	−	−				−	−	+	−	−	−	−	+	+							
6	−	−	+	−	+	−	+	−	−	−	−		+																
7	−	−	−	−	−	−	−	−	−	−				−	−	−	−	−	+	−	+	−							
8	−	−	−	−	−	−	−	−	−	−			−																
9	−	−	−	−	−	−	+	−	−	−	−		+																
10	−	−	+	−	−	−	+	−	−	−				−	−	+	−	−	+	−	−	−							
11	−	−	+	−	+	−	+	−	−	−	+		+																
12	−	−	−	−	+	−	+	−	−	−			+																
13	−	−	−	+	−	−	−	−	−	−			−																
14	−	−	+	−	−	−	−	−	−	−	−		−																
15	−	−	−	−	−	−	+	−	−	−	+/−		+/−																
16	−	−	−	+	−	−	−	−	−	−	−	−	−																
17	−	−	−	−	+	−	+	−	−	−	−		+																
18	−	+	−	+	−	−	−	−	−	−			−																
19	−	−	−	−	−	−	+	−	−	−			+/−																
20	−	−	−	−	−	−	−	−	−	−			+																
21	−	−	+	−	+	−	+	−	−	−	−		+																
22	−	−	−	−	−	−	−	+	−	+	−		−	−	−	−	−	−	−	+	−	−							
23	−	−	−	−	−	−	−	−	−	−	−		+																
24	−	−	−	−	−	−	−	−	−	−			+	+	+	−	−	−	+	+	−	−	−	−	−	−	+	−	−
25	−	−	−	−	−	−	−	−	−	−			−	−	+	−	−	+	−	−	−	−	−	−	−	−	−	−	−
26	−	+	−	−	−	−	+	−	−	−			+	−	+	−	+	+	+	+	+	−							
27	−	−	−	−	−	−	−	−	−	−	+		−																
28			−	−		−	−							+	+	−	−	−	+	−	−	−							
29	−	−	−	−	+	−	−	−	−	+				−	−	−	−	−	+	−	−	−							
30	−	−	−	−	−	−	−	−	−	−	−		+																
31	−	−	+	−	−	−	−	−	−	−	+		+																
32	−	−	−	−	−	−	−	−	−	−	+		−																
33	−	−	−	−	+	−	+	−	−	−	−		+/−																
34	−	−	−	−	−	−	+	−	−	−			−	−	−	−	−	−	+	−	−	−							
35	−	−	−	−	−	−	−	+	−	−			+/−	−	−	−	−	−	+	−	−	−							

Al, aluminum; Cem, cement; Co, cobalt; Cr, chromium; Cu, copper; Mo, molybdenum; Ni, nickel; Ti, titanium; V, vanadium; Zr, zirconium.

The “+” represents a positive test result, “−” a negative test result, and “+/−” an equivocal test result.

There were 18 patients with metal hypersensitivities diagnosed by testing who underwent primary TKAs with hypoallergenic components and had minimum 1-year follow-up. These patients had worse outcomes than matched controls (n = 18) without metal hypersensitivities undergoing primary TKAs with conventional TKA implants (Table 2). The 2 cohorts did not differ in any recorded preoperative measure. The postoperative changes however were lower in all categories for the metal-hypersensitivity cohort. Metal-hypersensitivity patients experienced significantly less improvement in KSS (36.1 vs 53.8, *P* = .03), Veterans RAND 12 physical component scores (7.6 vs 15.8, *P* = .04) and ROM (6.8° vs 20.7°, *P* = .03).

**Table 2:** Primary total knee arthroplasty matched cohorts.

Outcomes	Metal allergy cohort	No metal allergy cohort	*P* value
Preop ROM	114.6°	105.4°	.2
ROM change	6.8°	20.7°	.03^a^
Preop KSS FXN	52.5	50.8	.84
KSS FXN change	28.5	31.9	.37
Preop KSS	46.3	35.7	.08
KSS change	36.1	53.8	.03^a^
Preop VR-12 MCS	55.5	49.0	.09
VR-12 MCS change	0.21	5.1	.09
Preop VR-12 PCS	30.8	29.6	.64
VR-12 PCS change	7.6	15.8	.04^a^

FXN, function; MCS, mental component score; PCS, physical component score; Preop, preoperative; VR-12, Veterans RAND 12.

a Indicates statistical significance (*P* < .05).

There were 11 patients with test-confirmed metal hypersensitivity who underwent revision TKAs for an indication in addition to metal hypersensitivity using appropriate cemented hypoallergenic revision components (Table 3). Compared to matched controls who underwent revision TKAs for the same indication without a diagnosis of metal hypersensitivity, there were no differences between the cohorts with respect to preoperative measures with the exception that metal hypersensitivity patients had slightly greater preoperative ROM. Improvements after a revision surgery were typically worse in patients with metal hypersensitivities. KSS were actually worse after the revision surgery in the metal hypersensitivity cohort, which was significantly different compared to the revision cohort without metal hypersensitivities (-2.3 vs 14.1, *P* = .048).

**Table 3:** Revision total knee arthroplasty matched cohorts.

Outcomes	Metal allergy cohort	No metal allergy cohort	*P* value
Preop ROM	122.0°	111.1°	.02^a^
ROM change	3.6°	6.3°	.25
Preop KSS FXN	64.0	50.9	.18
KSS FXN change	−2.3	14.1	.05^a^
Preop KSS	51.9	43.1	.19
KSS change	14.9	24.5	.13
Preop VR-12 MCS	50.4	49.2	.82
VR-12 MCS change	−0.6	0.0	.44
Preop VR-12 PCS	32.4	28.2	.45
VR-12 PCS change	1.6	7.3	.18

FXN, function; MCS, mental component score; PCS, physical component score; Preop, preoperative; VR-12, Veterans RAND 12.

a indicates statistical significance (p < .05).

## Discussion

The management of metal hypersensitivities in the setting of joint replacement continues to be controversial. Without a consensus on the appropriate testing modality or indication for hypoallergenic implants, this patient population undergoing TKAs can be very difficult for orthopedic surgeons to manage. The current study highlights the inconsistency between different metal hypersensitivity testing modalities, which questions their reliability and utility for clinical decision-making. We also found that patients with metal hypersensitivities typically do worse after TKAs despite the use of appropriate hypoallergenic components than patients undergoing TKAs without metal hypersensitivities. Similarly, patients undergoing revision TKAs for metal hypersensitivity did not experience the same clinical improvement that patients without metal hypersensitivities experienced after revision TKAs.

The clinical relevance of metal hypersensitivities has been questioned in a number of previous studies [5,13,14,16,17,21]. Bravo et al. reviewed 127 patients undergoing TKAs, and 56 of them had metal hypersensitivities diagnosed by contact SPT [14]. SPT-positive patients did no worse after TKAs than SPT-negative patients, and within the group of SPT-positive patients, those receiving hypoallergenic implants had outcomes that were no different from those of patients receiving nonhypoallergenic implants. These findings led the authors to question the utility of SPT results in patients undergoing TKAs. The authors also identified a subgroup of patients with self-reported metal hypersensitivities but negative SPT. Interestingly, these patients had a higher incidence of arthrofibrosis after TKAs, which raised the question of whether patients' psychologic factors related to metal hypersensitivity contribute to worse outcomes after TKAs. Nam et al. reported on patients with self-identified metal hypersensitivities and found decreased satisfaction in TKA and THA patients with metal hypersensitivities [13]. Overall, 4% of their patients reported metal hypersensitivities, and 98% of them were female. Compared to nonmetal hypersensitive TKA patients, these patients had worse postoperative KSS, KSS function scores, and satisfaction. These findings led the authors to conclude that patients reporting metal hypersensitivities may have decreased satisfaction after TKAs and suggested that surgeons should consider counseling these patients preoperatively regarding this finding. Peña et al. specifically studied the psychologic consequences of metal hypersensitivity in 228 patients undergoing 245 TKAs [17]. Patients receiving hypoallergenic implants for metal hypersensitivity had significantly lower SF-12 mental and physical component scores, lower Western Ontario and McMaster Universities Arthritis Index scores, and lower Euro-quality of life measures than patients implanted with conventional components. The authors also worked with their psychiatry department to develop a psychologic distress scoring system based on psychiatric history, type of psychiatric pathology, and psychiatric drugs used. They found a higher incidence of severe psychologic distress in patients receiving hypoallergenic implants than in those receiving conventional implants (18.9% vs 4.4%, *P* = .041). While the authors questioned a possible correlation between psychologic distress and increased humoral immunity in the setting of metal hypersensitivity, they conceded that there is no clear explanation for worse outcomes and psychologic distress in patients with metal hypersensitivity receiving hypoallergenic implants.

Very limited literature has investigated the agreement between different metal hypersensitivity testing modalities within the same patients undergoing a joint replacement surgery. Thomas et al. reviewed the results of SPT, LTT, and histology tests in 25 patients with complications after TKAs using implants containing cobalt, chromium, and molybdenum metals [16]. Nine of the 25 patients had positive reactions to LTT tests for nickel and cobalt with negative SPT reactions, and 5 patients had positive SPT and LTT tests to nickel. There was a correlation analysis, but the results showed marked inconsistency between the tests used. Consistent with this finding, we also found a significant discordance between metal hypersensitivity testing modalities within the same patients. Our study appears to be the first to present a statistical correlation analysis between metal hypersensitivity tests. The discordance between test results is concerning given that there is currently no gold standard for metal hypersensitivity testing. When considering indications for hypoallergenic implants and a potential revision surgery for suspected metal hypersensitivity, orthopedic surgeons therefore need consistent test results to make informed clinical decisions. LTT has been supported as a more reliable, objective test [1,2,7], but more recent literature has failed to show a correlation between a positive LTT test and host immune response to the metal in synovial tissue surrounding the TKA implant [6].

The clinical relevance of metal hypersensitivity in TKAs will continue to be debated, and the current investigation only further questions the reliability of available metal hypersensitivity testing. Case reports and clinical series will support the clinical value of hypoallergenic implants [10,12,19,22,23], but the indications for these implants in primary and revision TKAs remain unclear. Matar et al. reviewed available literature and concluded that patients with self-reported metal hypersensitivities did benefit from hypoallergenic implants at short-term follow-up [4]. Our results show a clear inconsistency between different metal hypersensitivity testing modalities which can only question their utility in clinical decision-making. Additionally, patients with a diagnosis of metal hypersensitivity appear to do worse after both primary and revision TKAs even when the appropriate hypoallergenic implants are used. As extensive literature continues to emerge showing a relationship between patients' psychologic factors and clinical outcomes after TKAs [[24], [25], [26]], the current investigation adds to a body of literature suggesting a relationship between metal hypersensitivity and adverse clinical outcomes after TKAs. Whether metal hypersensitivity impacts patients' psychologic factors or a true immune response to metal implants remains unclear. While our clinical outcomes data do not support the use of hypoallergenic implants in the setting of metal hypersensitivity, we cannot recommend against the use of hypoallergenic implants given that our study did not include a subset of metal hypersensitivity patients receiving conventional implants. Ultimately clinical judgment should be used to determine which patients may benefit from hypoallergenic implants.

Limitations to our study are notable including the retrospective design and the small sample size. All contact SPT tests were performed at a single institution and over a large time period. As SPT techniques change, it is possible that results or interpretation of results could change with time. Additionally, more recent literature has strongly questioned the utility of contact SPT with potential sensitization over time [1,9]. Our study could be strengthened by a larger subset of metal hypersensitivity patients identified by testing modalities other than SPT. The current study is also limited by the inconsistency of testing within patients. Multiple testing modalities to more accurately diagnose metal hypersensitivity have been supported by previous literature, [15] and our study would have been strengthened if all subjects received the same types of testing for a more robust statistical analysis. TKA implants were not standardized in our study and were left to the discretion of the surgeon. The effects of different conventional or hypoallergenic implants on clinical outcomes were not controlled in this study. We were also unable to control for potential cross-reactivity between metal and cement hypersensitivity that could adversely affect clinical outcomes within the metal hypersensitivity revision cohort. The surgeons in our study did not use hypoallergenic implants except when indicated for metal hypersensitivity. Given this was infrequent, we cannot exclude potential effects of a surgeon’s comfort level with a different implant system on clinical outcomes.

## Conclusion

Different metal hypersensitivity tests produce conflicting results within patients and should be interpreted with caution when making clinical decisions regarding TKA implant choice and indications for a revision surgery. Given the potential for sensitization to metals and failure to simulate the immune system’s response to intra-articular metals, contact SPT has been proven less useful in current practice. Given that patients with metal hypersensitivity appear to do worse after both primary and revision TKAs, joint replacement surgeons should consider counseling patients on this finding before the surgery for appropriate informed decision-making. A future prospective study is required to better investigate the relationship between metal hypersensitivity and patients' psychologic factors and how this may affect clinical outcomes after TKAs.

## Conflicts of interest

Dr. D. Dennis receives royalties from DePuy, a Johnson & Johnson Company; is in the speakers' bureau of or gave paid presentations for DePuy, a Johnson & Johnson Company; is a paid consultant for Corin USA and DePuy, a Johnson & Johnson Company; has stock or stock options in Joint Vue; receives research support from DePuy, a Johnson & Johnson Company, and Porter Adventist Hospital; receives financial or material support from Wolters Kluwer Health–Lippincott Williams & Wilkins; and is in the editorial or governing board of Clinical Orthopaedics and Related Research, *Journal of Arthroplasty*, *Journal of Bone and Joint Surgery*, and *Orthopedics Today*. Dr. J. Jennings receives royalties from Total Joint Orthopedics; is a paid consultant for Total Joint Orthopedics and Xenex; has stock or stock options in Xenex; and receives research support from DePuy, a Johnson & Johnson Company. Dr. L. Kleeman-Forsthuber is a paid employee of Arthrex, Inc. and is an unpaid consultant for Corin. The other authors declare no potential conflicts of interest.

For full disclosure statements refer to https://doi.org/10.1016/j.artd.2022.09.016.

## Appendix A Supplementary data

Conflict of Interest Statement for Dennis

Conflict of Interest Statement for Bracey

Conflict of Interest Statement for Jennings

Conflict of Interest Statement for Johnson

Conflict of Interest Statement for Hegde

Conflict of Interest Statement for Kleeman-Forsthuber
