# Supplementary material for: Poor Correlation Among Metal Hypersensitivity Testing Modalities and Inferior Patient-Reported Outcomes After Primary and Revision Total Knee Arthroplasties
Source: Arthroplast Today. 2022 Oct 31;18:138–42. doi: 10.1016/j.artd.2022.09.016 (PMC9636001; doi:10.1016/j.artd.2022.09.016)
Supplement: Conflict of Interest Statement for Dennis [file mmc1.pdf]

INDIVIDUAL CONFLICT OF INTEREST STATEMENT

American Association of Hip and Knee Surgeons

(Adopted from the American Academy of Orthopaedic Surgeons disclosure statement)

The following form **must be filled out completely and submitted by each author (example, 6 authors, 6 forms). All items require a response. If there is no relevant disclosure for a given item, enter "*None.*"**

Manuscript Title: Poor correlation amongst metal allergy testing modalities and inferior patient reported outcomes after primary and revision total knee arthroplasty

1. Royalties from a company or supplier (The following conflicts were disclosed)
   DePuy, A Johnson & Johnson Company;

2. Speakers bureau/paid presentations for a company or supplier (The following conflicts were disclosed)
   DePuy, A Johnson & Johnson Company

3A. Paid employee for a company or supplier (The following conflicts were disclosed)
   None

3B. Paid consultant for a company or supplier (The following conflicts were disclosed)
   Corin USA; DePuy, A Johnson & Johnson Company

3C. Unpaid consultants for a company or supplier (The following conflicts were disclosed)
   None

4. Stock or stock options in a company or supplier (The following conflicts were disclosed)
   Joint Vue

5. Research support from a company or supplier as a Principal Investigator (The following conflicts were disclosed)
   DePuy, A Johnson & Johnson Company; Porter Adventist Hospital

6. Other financial or material support from a company or supplier (The following conflicts were disclosed)
   None

7. Royalties, financial or material support from publishers (The following conflicts were disclosed)
   Wolters Kluwer Health – Lippincott Williams & Wilkins

8. Medical/Orthopaedic publications editorial/governing board (The following conflicts were disclosed)
   Clinical Orthopaedics and Related Research; Journal of Arthroplasty; Journal of Bone and Joint Surgery; Orthopedics Today

9. Board member/committee appointments for a society (The following conflicts were disclosed)
   None

Each author must sign AND print or type his/her name, date and submit a separate form

In addition, one BLINDED Conflict of Interest form (no author names used) should be submitted per manuscript with all author disclosures.

Douglas Dennis	Douglas A. Dennis, MD	3/29/2022
Author Name (Print or Type)	Author Signature	Date
